# Can Liposomes Survive Inkjet Printing? The Effect of Jetting on Key Liposome Attributes for Drug Delivery Applications

**DOI:** 10.1007/s12247-022-09643-z

**Published:** 2022-05-24

**Authors:** Carolina Alva, Ivan Vidakovic, Barbara Lorber, Anna-Laurence Schachner-Nedherer, Manuel Zettl, Johannes Khinast, Ruth Prassl, Wen-Kai Hsiao

**Affiliations:** 1 Research Center Pharmaceutical Engineering GmbH, Graz, Austria; 2 Gottfried Schatz Research Center for Cell Signaling, Metabolism and Aging, Department of Biophysics, Medical University of Graz, Graz, Austria; 3 Faculty of Technical Chemistry, Chemical and Process Engineering and Biotechnology, Graz University of Technology, Graz, Austria; 4 Institute for Biomedicine and Health Sciences, Joanneum Research Forschungsgesellschaft mbH, Graz, Austria

**Keywords:** Inkjet printing, Liposomes, Lipid nanoparticles, Drug delivery system, Personalized medicine

## Abstract

**Purpose:**

Inkjet printing has the potential to enable novel personalized and tailored drug therapies based on liposome and lipid nanoparticles. However, due to the significant shear force exerted on the jetted fluids, its suitability for shear-sensitive materials such as liposomes, has not been verified. We have conducted a proof-of-concept study to examine whether the particle concentration and size distribution of placebo liposomes are affected by common inkjet/dispensing technologies.

**Methods:**

We have subjected three types of liposome-containing fluids (“inks”) to two different commercial dispensing/jetting technologies, which are relevant to most drug printing approaches. The liposome jetting processes were observed in real-time using strobographic imaging techniques. The phospholipid concentrations and particle size distributions were determined before and after jetting via enzymatic colorimetric and dynamic light scattering methods, respectively.

**Results:**

Our results have shown that the jetting dynamics of the liposome inks are well predicted by the established inkjet printing regime map based on their physical properties and the jetting conditions. Importantly, although significant shear forces were confirmed during jetting, the liposome concentrations and particle size distributions in the collected samples remain largely unaffected.

**Conclusion:**

These findings, we believe, provide the essential proof-of-concept to encourage further development in this highly topical research area.

## Introduction

First described by Bangham in 1965, liposomes are defined as small sphere-shaped artificial vesicles consisting of one or more phospholipid bilayers that surround a discrete aqueous space. Liposomes range in size from about 20 nm to several µm and spontaneously form in aqueous solution typically from nontoxic phospholipids along with cholesterol, which is used to tailor the permeability of the bilayers [1].

As phospholipids are major components of the plasma membrane, i.e., the outermost layer of mammalian cells, drug-encapsulating liposomes can serve as effective biocompatible tool for drug delivery [2]. By tailoring the lipid composition and the bilayer structure, liposomes can be designed to entrap either hydrophilic drugs within their aqueous interior or hydrophobic drugs within the hydrocarbon chain region of the phospholipid bilayer [3]. So far, more than 40% of the marketed nanocarriers are liposomal or liposomal/LNP complex formulations and there are currently 21 FDA/EMA approved products [4–9]. More recently, liposomes/LNP were established as gene delivery systems for targeted RNA-based therapy (Onpattro®) and vaccines to combat infectious diseases [10, 11]. Indeed, the recent vaccines developed by BioNTech/Pfizer and Moderna, which use LNPs as delivery vehicles for mRNA targeting the SARS-CoV-2 spike protein, represented a turning point in the fight against the COVID-19 pandemic [10, 12–14]. Based on this success, and considering the urgent clinical need for new therapies, it can be expected that more liposomes/LNP-based pharmaceutical products will be launched by the pharmaceutical industry in the near future [15–17].

As the versatility of liposome/LNP drug delivery systems has been demonstrated for various drug products and multiple administration routes, new technologies of liposome manufacturing and handling will be needed [18–21]. Whereas liquid dosage forms remain the primary applications for liposome/LNP carriers, novel printed solid dosage forms have gained increased research and development interests in recent years. For example, a recent study has shown that hydrogels containing PEGylated liposomal doxorubicin can be printed using a semi-solid extrusion-type 3D bioprinter, with drug release from the patches being dependent on the shape of the printed patches [22]. Whilst the study demonstrated the viability of liposomes embedded in hydrogel patches, it is not yet clear to what extent free liposomes, due to their fragile nature, can indeed survive post-processing steps [23, 24]. Furthermore, consolidating powders via inkjet printing of binder liquids is a key enabling technology for many 3D printing approaches for pharmaceutics [25], including the first FDA-approved 3D-printed drug product Spritam (levetiracetam) for the treatment of epilepsy [26]. It is conceivable that liposome/LNP-based formulations could be jetted as functional binders in such applications.

**Fig. 1:**
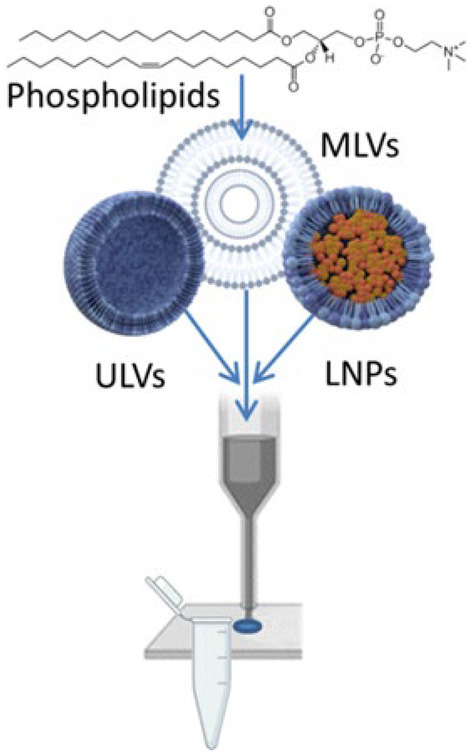
Liposomes, assembled from phospholipids to form multilamellar vesicles (MLV), unilamellar vesicles (ULV) or lipid nanoparticles (LNPs), may be jetted and deposited to form tailored drug doses

Nozzle-based inkjet printing typically uses thermally or mechanically induced impulses to generate extremely high shear rates in fluids in order to produce micro- to pico-liter sized droplets. Although a study has shown that potentially fragile mammalian nerve cells could survive such stress levels during printing [27], it is not yet certain that such outcome could be expected when printing fluids containing liposomes. We have conducted a preliminary proof-of-concept case study, shown conceptually in Fig. 1, that aims to understand whether preformed liposomes can be printed using piezoelectric printers with minimal impact on their key attributes, specifically concentration and particle size distribution, which are relevant to their pharmaceutical applications.

## Methods and Materials

### Materials

1-Palmitoyl-2-oleoyl-glycero-3-phosphocholine (POPC) was purchased from Avanti Polar Lipids, Inc. (Alabaster, AL, USA). 5-Cholesten-3β-ol (Cholesterol) (Sigma Grade > 99%) and absolute ethanol were ordered from Sigma Aldrich (St. Louis, MO, USA) and N-(2-Hydroxyethyl)piperazine-N′-(2-ethanesulfonic acid) (HEPES) and sodium hydroxide pellets from Carl Roth, GmbH + Co. KG (Karlsruhe, Germany).

### Liposome Formation

Two different techniques were used for the preparation of liposomes: dry film rehydration and microfluidics. For the dry film rehydration technique, appropriate amounts of POPC and cholesterol were dissolved in a mixture of chloroform–methanol 2:1 (vol/vol). The lipid film was obtained by evaporating the organic solvents with a stream of nitrogen, followed by drying overnight in a vacuum chamber. The dried film was rehydrated with 1 mL 10 mM Hepes buffer, pH 7.4, to obtain a final phospholipid concentration of 30 mg/mL and a cholesterol content of 30 mol%. The addition of the liquid phase causes the spontaneous formation of multilamellar vesicles (MLV) in the size range of a few microns. Downsizing of liposomes was performed at room temperature by eight one-minute rapid vortex-mixing cycles at 15-min intervals. The mixing was followed by 21 steps of size extrusion through a 100 nm polycarbonate membrane filter (Whatman Inc., Clifton, NJ) in a Mini Extruder (Avanti Polar Lipids, Alabaster, AL), resulting in unilamellar vesicles (ULV) of a very narrow size range roughly corresponding to the nominal membrane pore size.

The microfluidic-based liposome preparation was performed by dissolving the same amounts of POPC and cholesterol as described above, in 5 mL absolute ethanol. The lipid-ethanol fraction was mixed with 5 mL 10 mM Hepes buffer, pH 7.4, in a 1:1 volume ratio within the microfluidic cartridge of the NanoAssemblr Benchtop system (Precision NanoSystems, Vancouver, BC). The total flow rate of the liquids was fixed to 5 mL/min resulting in ULVs with an approximate phospholipid concentration (PC) of 3 mg/mL. The preparation was followed by immediate centrifugation using Vivaspin 20 tubes with a 100-kDa cut-off integrated membrane (Satorius AG, Goettingen, GE). This step is essential for two reasons: First, to remove the high concentration of organic solvent (in our case about 50% ethanol), which destabilizes the system and can lead to particle aggregation. Second, to increase the concentration of liposomal particles to approximately 30 mg/mL. Thus, the same sample properties were obtained for both preparation techniques.

### Particle Size Determination

Particle size distribution was measured using a Zetasizer 3000HS (Malvern-Panalytical Ltd., Malvern, UK) after every critical step: rehydration, extrusion, mixing, centrifugation, pre- and post-printing. The measurements were performed at room temperature, and all used samples were diluted to a final phospholipid concentration of 0.3 mg/mL. The uniformity or the width of the sample particle size distribution was expressed using the polydispersity index (PDI) within the range from 0 (completely uniform) to 1 (heterogeneous sample).

### Lipid Content

To ensure approximately the same amount of lipids within each sample, before and after printing, the phospholipid concentration (PC) was determined using the Phospholipid FS assay (DiaSys, Holzheim, Germany). The enzymatic assay relies on colorimetric determination of the reaction product between choline-containing phospholipids and the diagnostic reagent. Along with the enclosed phospholipid standard solution, each sample was pipetted onto the 96-well microplate in the absolute amount of 25 µL. A Clariostar plate reader (BMG LABTECH, Ortenberg, Germany) was used to inject the volumes of 160 µL and 40 µL of enclosed reagents, followed by the single wavelength absorbance measurement at 570 nm, according to the manufacturer´s protocol.

### Surface Tension and Viscosity

The surface tension was measured by the optical pendant drop method using a nozzle tip diameter of 1.8 mm with the instrument EasyDrop (KRÜSS GmbH, Hamburg, Germany). It consists of forming a pear shape drop hanging from a needle right before falling and correlating the drop curvature to the surface tension via the Laplace equation. The risk of sedimentation of MLV in the needle was reduced by rigorously shaking the sample before measurements. The MLV ink was rapidly loaded and measured immediately to minimize errors. EXTR-ULV and MF-ULV samples were measured without any precautions.

The viscosity of the samples was determined with the MCR 302 rotational rheometer and a cone plate geometry (AntonPaar, Graz, Austria). The shear rate was linearly increased from 1–100 s^−1^ (50 data points). The measurements were performed at 20 ºC.

### Jetting and Ink Sample Collection

Aspects of the liposome ink jetting and printing dynamics were studied using two different inkjet systems. Specifically, a glass capillary-based dispensing system (sciFLEXARRAYER S3, Scienion AG, Berlin, Germany) was used to monitor the fluid movement in the nozzle leading to jet and drop formation, and a research inkjet printer (PixDRO LP50, SÜSS MicroTec SE, Garching, Germany) was used to verify the feasibility of scaling up/down to the modular, industrial inkjet print heads.

Preformed liposome samples (MLV, EXTR-ULV and MF-ULV), which were prepared as described above at a comparable lipid concentration of about 30 mg/mL, were used as inks without further treatment.

All jetting and printing tests were carried out in triplicate at ambient conditions (20 °C and 45% relative humidity (RH)). Each post-jetted ink sample was pipetted and diluted with 10 mM Hepes buffer, pH 7.4, to 50 and 10 µL/ of total volume for further characterization. Pure buffer, as well as aliquots of the pre-jetted inks, were analyzed as controls. The particle size distribution of the liposomes has been determined by photon correlation spectroscopy (dynamic light scattering) taking into account intensity, volume, and number distributions. The data are presented as Z-average ± SD. For the statistic evaluation of changes in particles size before and after printing, a Wilcoxon signed-rank test was used. Statistical significance was assumed at *p* < 0.05. Statistics was performed using the program SPSS Statistics (IBM Corp. Version 27.0. 1.0, Armonk, NY).

#### Jetting via the Scienion Glass Capillary

**Fig. 2:**
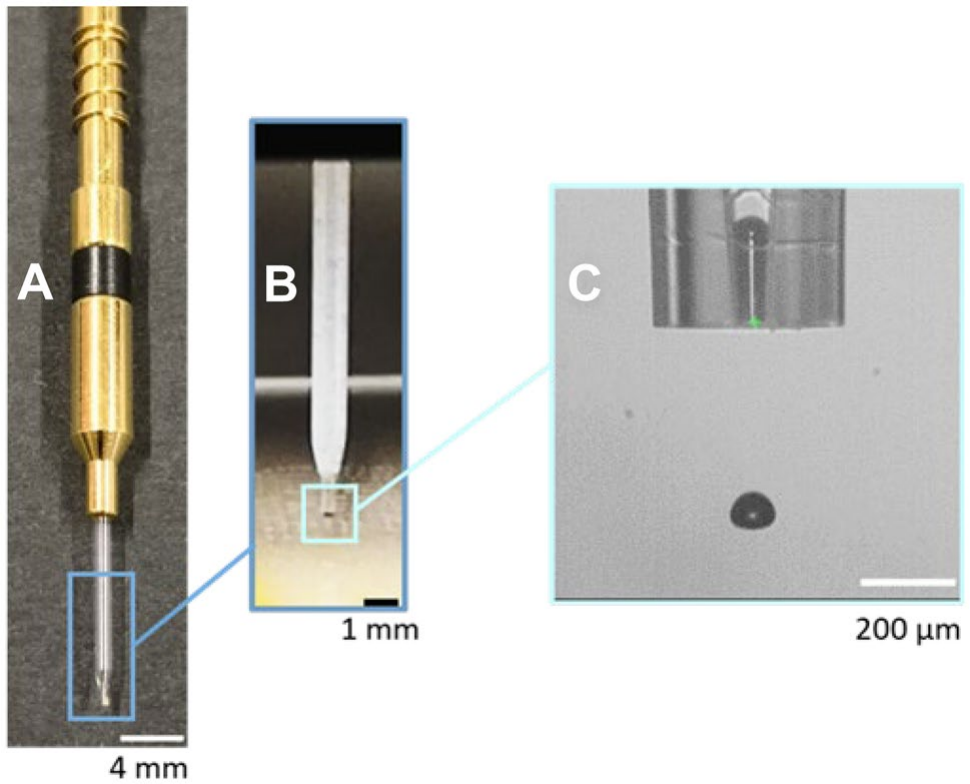
A Scienion piezo/capillary dispensing device (**A**) in spotting (**B**) and jet/drop monitoring (**C**) modes

As shown in Fig. 2, a Scienion dispensing device (PDC90/P-2050), consisting of a glass capillary with an opening of 90 µm in diameter surrounded by a piezo actuator, was installed in the sciFLEXARRAYER S3 system. The device was pre-filled with a system fluid (0.22 μm filtered and degassed de-ionized water), which was used to aspirate around 25 µl into the glass capillary. An electric pulse with amplitude of 175 V and 255 µs in duration was applied to the piezo actuator at 750 Hz frequency to eject drops acoustically at picoliter scale from the nozzle. A camera-strobe system was used to monitor the liquid meniscus movement, as well as jet and drop formation dynamics.

The ejected liposome ink drops were collected in a centrifuge vial positioned directly below the dispensing device. The jetting was stopped when the interface between the system fluid and the liposome ink became visible above the throat of the capillary in order to prevent contamination of the collected ink sample.

#### Jetting via an Industrial Print Head

**Fig. 3:**
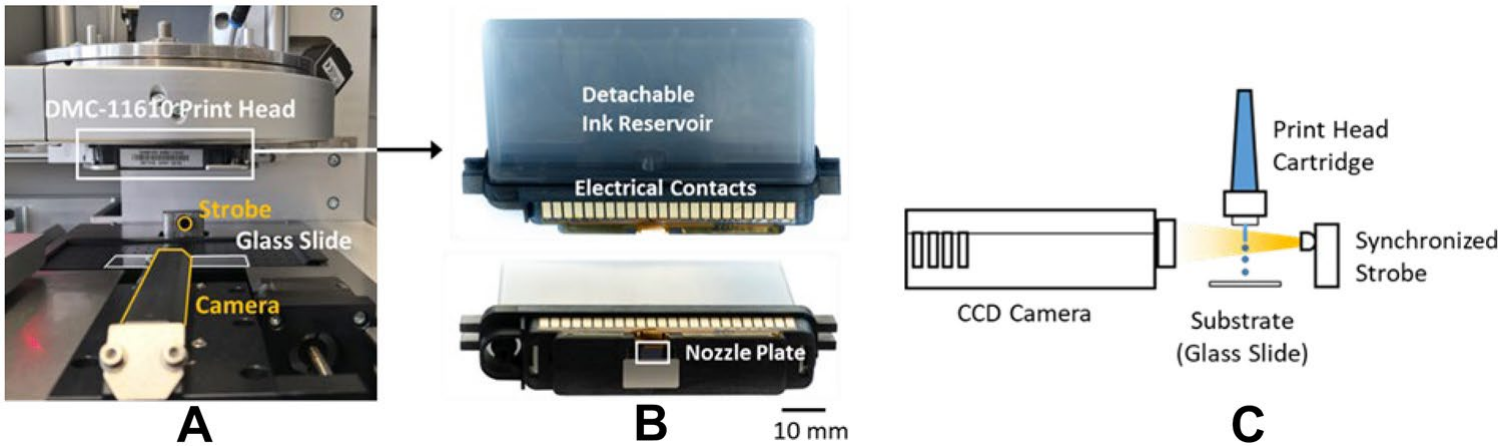
The Dropview station in the PixDRO LP50 printer (**A**), details of the Dimatix DMC print head cartridge (**B**), and the schematic of jet/drop monitoring (**C**)

Once the basic jet-ability of a liposome ink was established using the Scienion dispensing device, the same ink formulation was transferred to a cartridge print head (DMC-11610, FUJIFILM Dimatix, USA) installed in the PixDRO LP50 printer as presented in Fig. 3. An ink volume of 900 µl was loaded into the cartridge reservoir and a basic jetting waveform was developed using the built in dropwatcher (Dropview) system.

Approximately 50 μl of ink was jetted onto a glass slide positioned directly at the Dropview station. The deposited ink was collected using an Eppendorf Research Plus 0.5–10 μl pipette (Eppendorf SE, Hamburg, Germany) immediately after jetting in order to minimize errors due to evaporation/sedimentation. The pipette tip was submerged and stirred in the ink pool before pipetting. In general, less than 10% of the deposited ink were pipetted. The collected ink was immediately diluted with Hepes buffer solution for further analysis.

## Results and Discussion

### Jettability Evaluation Outcome

**Fig. 4:**
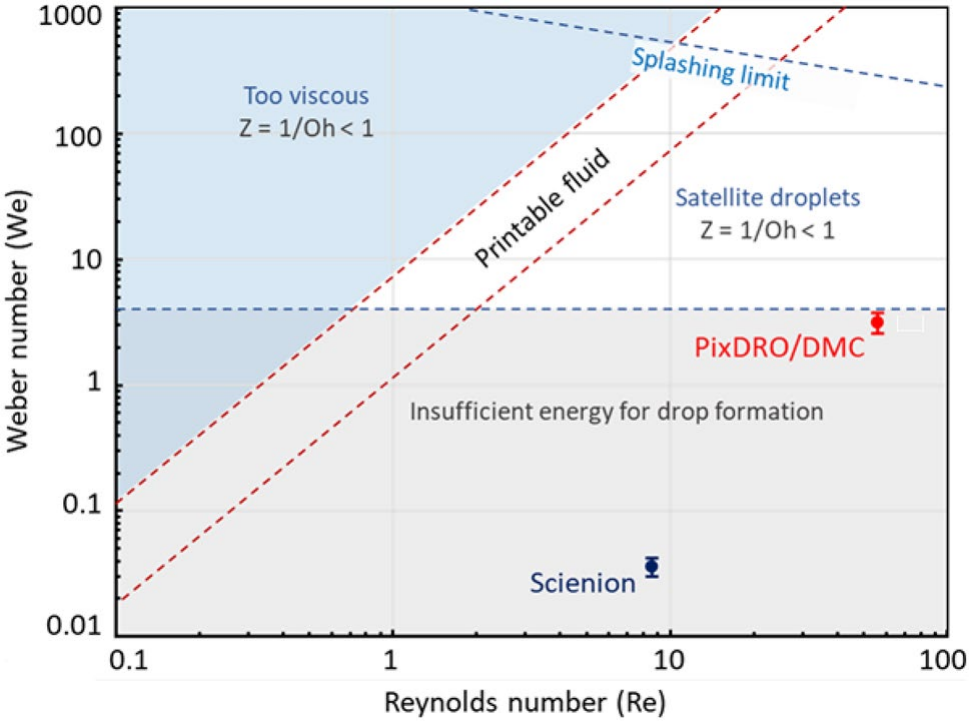
The parameter ranges of our inkjet experiments using the Scienion (blue point in the graph) and PixDRO/DMC (red point in the graph) systems overlaid onto the general inkjet printability regime (adapted from [18])

There are two main criteria for inkjet-ability for any ink formulation: ejection dynamics and nozzle availability. In order to eject an ink from a nozzle, the drive energy supplied by the piezo actuator needs to overcome the dissipative (viscosity) and elastic (surface tension) forces in the ink formulation. Ideally, the ejected jet should break up cleanly into a single droplet with sufficient velocity to ensure accurate drop placement on surface. However, when the fluids have a higher surface tension, additional, undesired satellite droplets are often formed. The regions of appropriate ejection dynamics can be empirically defined by non-dimensional groups of Reynolds (Re), Weber (We), and Ohnesorge (Oh or 1/Z) numbers as shown in Fig. 4 [28].

In general, for liposome dispersions in aqueous solution, the surface tension predominantly depends on the lipid concentration and the temperature. For unstressed dipalmitoylphosphatidylcholine (DPPC) liposomes at low concentrations in the gel phase (i.e., below ~ 40 °C), the dynamic surface tension was reported to be close to water at ~ 70 mN/m [29, 30]. At higher lipid concentrations (e.g., greater than 20 mg/mL DPPC, 33 °C), the surface tension could decrease to ~ 30 mN/m, whereas the viscosity remains Newtonian at ~0.95 mPas [31]. For the liposome inks applied in this study, i.e., multilamellar liposomes (MLV), microfluidic-synthesized unilamellar liposomes (MF-ULV) and extruded unilamellar liposomes (EXTR-ULV) composed of 1-palmitoyl-2-oleoyl-glycero-3-phosphocholine (POPC) and cholesterol with a phospholipid concentration of about 30 mg/mL measured at 20 °C, the results of the surface tension measurements are well within this range. For MF-ULV, the surface tension was 57.86 ± 0.6 mN/m (n = 10), being lower than for the other two samples, MLV and EXTR-ULV with 72.03 ± 0.6 and 72.64 ± 0.11 mN/m (*n* = 10), respectively. Likewise, the drop volume achieved for MF-ULVs (24.13 ± 0.23 µl) was smaller compared to MLVs (30.14 ± 0.23 µl) and EXTR-ULVs (30.63 ± 0.24 µl). The lower surface tension determined for MF-ULV can be explained by the residual amounts of ethanol present in MF-ULV ink preparations. Concerning viscosity, the liposome inks showed a Newtonian fluid behavior, but a somewhat higher viscosity of 1.2–1.3 mPas as expected, being highest for the MLV sample.

Based on the associated ranges of Re and We numbers in the inkjet regime map shown in Fig. 4, we have expected difficulties in ejecting the liposome inks using the Scienion system. Therefore, the maximum impulse setting would be needed for the associated experiment. Nevertheless, both jet consistency and drop speed from the Scienion capillary could still be sub-optimal. On the other hand, the regime map indicated that the liposome inks could indeed be jetted using the PixDRO printer with the DMC print head, although satellite droplets were likely to be prevalent. These predictions were confirmed in the actual jetting experiments.

**Fig. 5:**
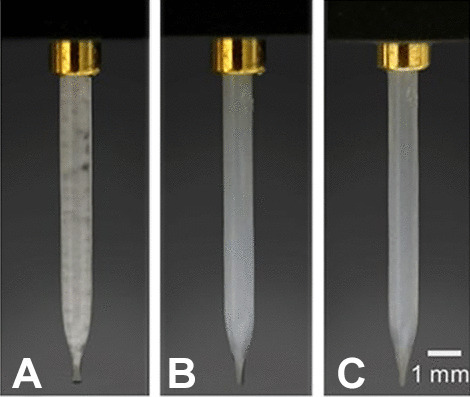
Scienion piezo-dispense capillaries loaded with polydisperse MLV (**A**), 170 nm MF- ULV (**B**), and 150 nm EXTR-ULV (**C**) inks. The image backgrounds have been edited to allow better visualization of the differences in agglomeration within the capillaries

The MLV ink was, as shown in Fig. 5A, the most challenging ink due to the significant amount of agglomerations observable in the glass capillary. Although drop ejection was in general possible, blockage due to particle settlement in the nozzle throat occurred after a few seconds if jetting was paused or interrupted. No such settlement issue was observed when jetting microfluidic-synthesized liposomes (MF-ULV) or extruded liposomes (EXTR-ULV) inks, as shown in Fig. 5B, C, respectively.

**Fig. 6:**
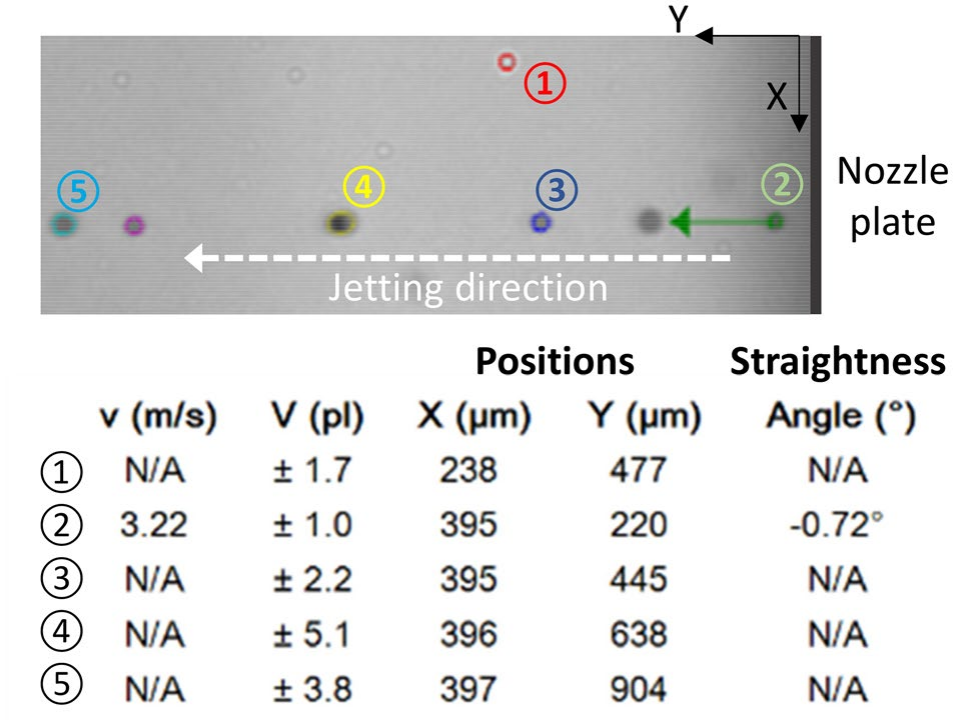
An example of Dropview analysis results (150 nm EXTR-ULV ink) using the Pixdro LP50 dropwatcher system

The jetting of MLV, EXTR-ULV, and MF-ULV inks using the DMC print heads in the PixDRO LP50 printer yielded similar observations. From the images captured by the PixDRO’s Dropview system, as shown in Fig. 6, we could estimate key process attributes such as drop velocities, drop sizes, drop positions, and jet straightness. Specifically, the drop size, including main and satellite drops, ranged from 1 to 6 pl with an average speed around 3 m/s. Furthermore, all three liposome inks were prone to satellite generation. However, as the smaller nozzles in the DMC heads (20 µm vs. 90 µm in the Scienion capillary) may be more susceptive to partial blockage, the root cause cannot be solely attributed to ink properties (e.g., surface tension and viscosity).

**Fig. 7:**
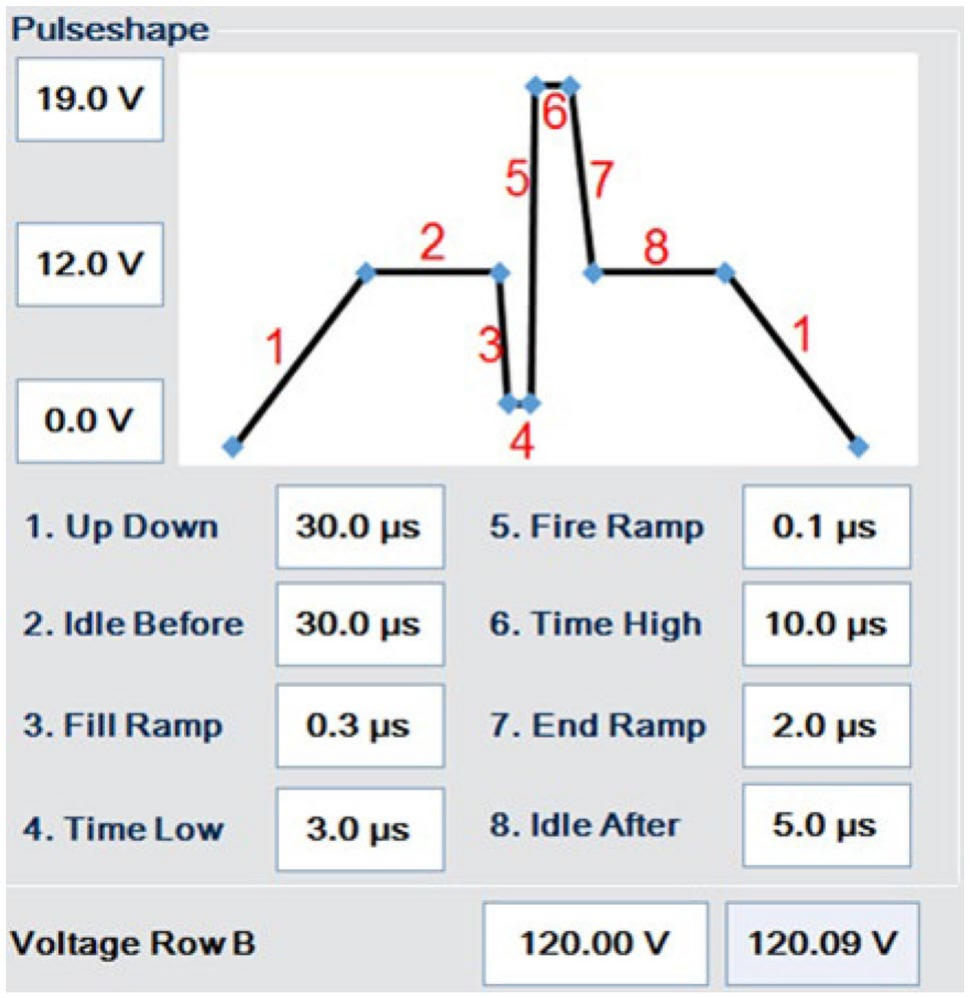
Piezo drive waveform used for jetting the MLV, EXTR-ULV and MF-ULV inks

It is worth noting that the jetting behavior can be improved, e.g., reducing satellite generation, by optimizing the drive waveform delivered to the piezo actuators. This is typically done in an iterative fashion with further ink formulation development. As the current work is intended as a proof-of-concept (POC) study, only the default waveform, as shown in Fig. 7, with basic adjustments of pulse amplitude and duration was used.

### Estimation of Shear Rate During Jetting

The shear rate γ, experienced by the liposome formulations during ejection, was estimated based on the method proposed by Dybowska-Sarapuk et al. [32]

1 $$\gamma \ge \frac{{V}_{\mathrm{drop}}}{0.166 d}$$

where *V*_drop_ and *d* are droplet velocity (average in our cases) and nozzle diameter, respectively. Based on the average drop velocities measured, we estimated the minimal shear rates the inks were subjected to within the Scienion capillary and the DMC cartridge head to be approximately 9 × 10^3^ s^−1^ and 9 × 10^5^ s^−1^, respectively. Both estimated values are consistent to and within the ranges of those reported in literatures [32, 33]. It is expected that the estimated shear rate in the Scienion capillary is much lower than that in the DMC cartridge, primarily due to the larger nozzle size (90 µm vs. 21.5 µm) and lower jet speed (0.13 m/s vs. 3.22 m/s). However, it has been shown that it is possible to achieve equivalent shear rate in the capillary-based jetting device [27]. Furthermore, the stress condition experienced by fluids within a capillary could considerably differ from that in an industrial inkjet print head. For example, the shear duration could be notably greater due to the more gradual narrowing of the fluid chamber toward the nozzle. Therefore, it is important to examine the jetting outcomes using both printing technologies.

### Analysis Outcome of the Pre- and Post-jetted Liposome Inks

The influence of the printing process on liposomal particles was evaluated over two preparation methods and at critical preparation stages. The results of the size and uniformity for the used formulations were summarized in Table 1. It is important to note that the particle size of the ULVs (EXTR-ULV and MF-ULV) as well as their uniformity has not been significantly changed during the printing process (*p* = 0.10 and *p* = 0.11 for EXTR-ULV and MF-ULV, respectively) despite of the high shear forces within used devices. The MLV ink, on the other hand, tends to agglomerate with time to form aggregates up to 20 µm in size. This presents a challenge for characterization as such aggregate size can breach the upper limit of suitability for the dynamic light scattering technique. As expected, fast sedimentation indicates the presence of large emulsified particles, resulting in non-reproducible size measurement values. Accordingly, MLVs that tend to aggregate could not be printed with the DMC cartridge due to the very small nozzle size of the device.

**Table 1:** Particle size distribution of the three liposomal preparations at different steps of the manufacturing process, expressed as mean particle size (MPS) and polydispersity index (PDI) with standard deviations (SD) for *n* ≥ 3. T1, after preparation; T2, prior to printing; and T3, after printing

**Scienion dispensing device**										
		**MLV**			**EXTR-ULV**			**MF-ULV**		
		**T1**	**T2**	**T3**	**T1**	**T2**	**T3**	**T1**	**T2**	**T3**
** MPS [nm]** ± **SD**		3285.5 ± 247.2	3111.1 ± 183.6	2645.4 ± 110.5	144.8 ± 0.5	140.8 ± 0.6	142.8 ± 0.4	172 ± 0.1	172.6 ± 0.8	174.8 ± 0.9
** PDI** ± **SD**		1.0	1.0	1.0	0.121 ± 0.007	0.112 ± 0.008	0.111 ± 0.021	0.086 ± 0.012	0.091 ± 0.009	0.095 ± 0.003

**PixDRO/DMC cartridge print head**										
					**EXTR-ULV**			**MF-ULV**		
					**T1**	**T2**	**T3**	**T1**	**T2**	**T3**
** MPS [nm]** ± **SD**					162.7 ± 0.7	159.3 ± 0.5	159.8 ± 0.4	157.9 ± 0.4	159.5 ± 0.9	160.5 ± 1.1
** PDI** ± **SD**					0.168 ± 0.005	0.175 ± 0.021	0.179 ± 0.015	0.105 ± 0.009	0.093 ± 0.013	0.091 ± 0.01

To ensure that the printing process does not affect only quality but also quantity of the liposomal particles, a phospholipid assay was used to quantify the content of PC before and after printing. Due to the small amounts of ink sample collected, dilutions were required to increase the sample volumes and facilitate content measurement. The dilution factors were determined based on the average drop sizes and the estimated number of drops collected (2 × 10^5^ and 2 × 10^7^ drops for the Scienion and PixDRO/DMC experiments, respectively). Due to the variations in both factors, there are notable deviations of the concentration values of the printed samples (T2) compared to the samples before printing (T1), as seen in Table 2. To evaluate the reproducibility of the printing process, three independent jetting experiments with two dilution factors (*n* = 6) have been performed, which yielded similar mean values but with a considerable standard deviation (Table 2). Indicated by the significantly higher deviations of the T2 PC values (*p* < 0.05), i.e., greater SD, it appears that the extruded liposomes might be less reliably transported by inkjet printing compared to the microfluidics processed liposomes. However, it is difficult to confirm these observations currently as the low sample volumes (hence, the high uncertainty in the associated dilution factors) prevented us from developing a clear correlation between the printing devices and the liposome inks.

**Table 2:** Phospholipid content (PC) of the liposomal preparations in the initial sample before printing (T1) and after printing (T2). The data are presented as mean ± SD (*n* = 6, representing three printing trials and two dilution factors)

**Scienion dispensing device**						
	**MLV**		**EXTR-ULV**		**MF-ULV**	
	**T1**	**T2**	**T1**	**T2**	**T1**	**T2**
** PC [mg/mL] ± SD**	35.81	30.35	30.56	17.75	32.91	21.27
	± 1.22	± 6.17	± 0.35	± 10.62	± 1.32	± 4.30

**PixDRO/DMC cartridge print head**						
			**EXTR-ULV**		**MF-ULV**	
			**T1**	**T2**	**T1**	**T2**
** PC [mg/mL] ± SD**			33.11	41.99	20.94	24.74
			± 0.88	± 14.75	± 0.42	± 4.89

Another interesting aspect for this study was to examine whether inkjet printing can be used as a method to resize liposomes for drug delivery. Typically, liposomes are produced by lipid film rehydration that results in a heterogeneous mixture of MLVs in a micrometer size range. Such MLVs are generally too big for drug delivery applications and require further resizing and homogenization steps. The most popular approach is a size extrusion procedure, in which the MLVs are passed through polycarbonate filter membranes of defined pore size yielding an almost monodisperse solution of ULVs of controlled average size that depends on the nominal pore size of the filter membrane used for the extrusion process [34]. It was hoped that by applying highly controlled shear conditions within the ink chamber and nozzle, a control over the liposome size could be exerted. By doing so, ink jetting of preformed MLVs was considered as option to replace the extrusion steps in the preparation protocol for ULVs. However, as mentioned before, MLVs could not even be printed by the DMP device because of larger aggregates clotting the nozzle. Using the Scienion capillary dispenser, the preliminary jetting results have shown negligible changes in particle size distribution and polydispersity, suggesting that the shear forces applied may not be sufficient to homogenize MLVs. On the other hand, these results support our findings that liposomes can be transported and printed by jetting in a non-destructive manner. Thus, inkjet printing technologies seem to be suitable for the delivery of novel lipid-based drug dosage forms. Once optimized, we believe that this approach could enable transferring and delivering of liposome/LNP entrapped drug substances with highest accuracy in a controlled manner [35].

## Conclusions

ULVs, which meet the physicochemical characteristics required for drug delivery, remained almost unaltered in concentration and particle size distribution after jetting. Therefore, our results suggest that, with appropriate ink formulations, the processing stress of inkjet printing should not adversely affect the viability of liposomal delivery systems. Furthermore, the synthesis of our liposomes follows two standardized procedures and the jetting parameters used are lightly modified, default values of the respective dispensing/printing systems, i.e., standard pulse waveforms with modified amplitudes. The fact the un-optimized processes we used were sufficient to allow successful jetting of liposomes demonstrates the potential of this approach.

We would like to stress the preliminary nature of our findings, as indeed more elaborated approaches could be used to quantify the liposome viability after printing further. For example, scanning electron microscopy (SEM) and cryogenic transmission electron microscopy (Cryo-TEM) are viable approaches to investigate the laminar structure, hence the functional integrity of the liposomes [36, 37]. However, we believe that such additional confirmation could be achieved by the intended follow-up of this feasibility study, which will aim to encapsulate model drugs, including chemotherapeutics or biomolecules, into the ULVs. By determining the drug “leakage” after printing, the stability of such ULV-drug systems could be effectively quantified. The ability to inkjet-printed intact liposomes of different composition and payload could create new opportunities for a combination therapy paving the way for patient-customized drug delivery systems in precision medicine.
